# Carbon-Carbon Double Bond and Resorcinol in Resveratrol and Its Analogues: What Is the Characteristic Structure in Quenching Singlet Oxygen?

**DOI:** 10.3390/biom9070268

**Published:** 2019-07-09

**Authors:** Qingjun Kong, Xueyan Ren, Jianrui Qi, Jia Yu, Jun Lu, Shuo Wang

**Affiliations:** 1 State Key Laboratory of Food Nutrition and Safety, Tianjin University of Science & Technology, Tianjin 300222, China; 2 Shaanxi Engineering Laboratory for Food Green Processing and Safety Control, and Shaanxi Key Laboratory for Hazard Factors Assessment in Processing and Storage of Agricultural Products, College of Food Engineering and Nutritional Science, Shaanxi Normal University, Xi’an 710119, China; 3 College of Food Science, Southwest University, Chongqing 400700, China; 4 School of Science, and School of Interprofessional Health Studies, Faculty of Health & Environmental Sciences, Auckland University of Technology, Auckland 1142, New Zealand

**Keywords:** Resveratrol analogues, singlet-oxygen quencher, resorcinol, UHPLC-QTOF-MS^2^, UHPLC-QQQ-MS^2^, hydroxyl groups

## Abstract

Stilbenes, particularly resveratrol and resveratrol dimers, could effectively quench singlet oxygen (^1^O_2_). It was reported that both resorcinol and carbon-carbon double bond quenching ^1^O_2_ can participate in the mechanism. However, it is still not clear which structure plays a dominant role in quenching ^1^O_2_. To investigate the characteristic structure in the mechanism of quenching ^1^O_2_, the resveratrol, pterostilbene and piceatannol quenching ^1^O_2_ abilities were compared by UHPLC-QTOF-MS^2^ and UHPLC-QQQ-MS^2^. Results showed that catechol, carbon-carbon double bond and resorcinol participated in the quenching of ^1^O_2_. Catechol ring plays a leading role in the mechanism, and the contribution of the structures in quenching ^1^O_2_ activity are as follows: catechol ring > carbon-carbon double bond > resorcinol ring, which is supported by the calculation of energy. Our findings will contribute to the future screening of stilbenes with higher activity, and those stilbenes may have great therapeutic potential in ^1^O_2_-mediated diseases.

## 1. Introduction

As mediators of oxidative stress, reactive oxygen species (ROS), including superoxide radical anion (O_2_^•−^), hydroxyl radical (·OH), singlet oxygen (^1^O_2_) and hydrogen peroxide, have been regarded as the main reason leading to many diseases [1]. As the first excited state ^1^Δ_g_ of molecular oxygen, ^1^O_2_ is considered as one of the most active classes involved in chemical and biochemical reactions, since it can easily react with a large number of biological molecules, such as DNA, proteins and lipids [2]. ^1^O_2_ mediated DNA damage can lead to neurological disorders like Alzheimer’s and Parkinson’s disease, and even increased risk of cancer [3]. Furthermore, ^1^O_2_ ias reported to be closely associated with the occurrence of xeroderma pigmentosum, a rare skin disorder [4,5]. The generation of ^1^O_2_ is achieved by endogenous photosensitizers such as chlorophyll or riboflavin absorbing energy from both ultraviolet (UV)-A and UV-B as well as visible light then transferring the energy to the oxygen molecule [6]. Numerous studies have emphasized the hazards caused by ^1^O_2_ to human health [7,8]. 

Stilbenes are a class of natural phytoalexins found in plants (especially grapes) and are well-known for their antioxidant activity, exhibiting various biological activities such as cardioprotection, neuroprotection, anti-diabetic properties, depigmentation, anti-inflammation, and cancer prevention and treatment [9]. Stilbenes have a common backbone stilbene structure but differ in substituents on the ring [10]. Structural complexity and diversity of stilbenes lead to the difference of its biological activity. Ohguchi et al. (2003) suggest that the carbon-carbon double bond in the stilbene skeleton is critical for the inhibitory effects against murine tyrosinase activity [11]. Literature showed that the antioxidant activity was involved in the number and position of hydroxyl groups. Murias found that 3,3′,4,4′,5,5′-hexahydroxystilbene exerted a more than 6600-fold higher antiradical activity than resveratrol and its two other analogues [12]. Similarly, Kotora et al. speculated that releasing of the proton from OH group could be the main mechanism responsible for the antioxidant activity of studied (Hydroxyphenyliminomethyl) phenols [13]. In addition, methoxylation of hydroxyl has been reported to significantly improve the anti-tumor potential of stilbene compounds [14].

The activity of stilbene quenching ^1^O_2_ has not caught deserved attention for a long time in the past, until pallidol, a resveratrol dimer, was discovered as a selective ^1^O_2_ quencher by He et al. [15]. Additionally, it has been reported that vitisin A from *Vitis chunganeniss* shows the activity of selective ^1^O_2_ quenching [16]. Subsequently, stilbenes like chunganenol, laetevirenols F and laetevirenols G have been determined to be potent ^1^O_2_ quenchers by electron paramagnetic resonance experiments [17,18]. Later, scirpusin A, a hydroxystilbene dimer from Xinjiang wine grape, was reported as an effective singlet oxygen quencher [19]. In the further investigation of the activity of stilbenes quenching ^1^O_2_, many works so far have focused on its mechanism. Some hold the view that resorcinol ring could be oxidized to quinone when resveratrol quenches ^1^O_2_ [20]. Others propose that the mechanism of resveratrol ^1^O_2_ quenching is mainly because of the carbon-carbon double bond based on nuclear magnetic resonance (NMR) data [21]. In addition, the mechanism of resveratrol dimers quenching ^1^O_2_ has been investigated by UHPLC-QTOF-MS^2^ in our previous studies, suggesting that resorcinol ring and carbon-carbon double bond both participate in quenching ^1^O_2_ [22]. Nevertheless, which structure plays a more important role in the mechanism of quenching ^1^O_2_ is still not clear.

**Figure 1:**
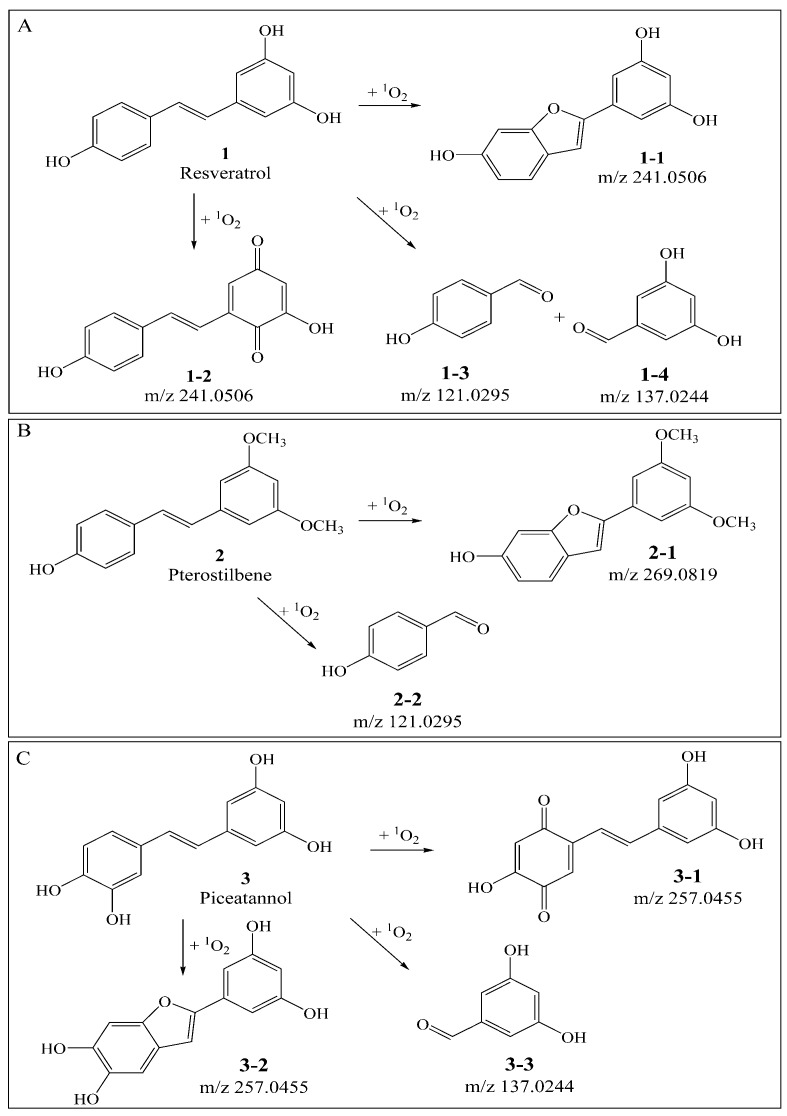
(**A**): Proposed mechanism of resveratrol quenching ^1^O_2_ (**1**: resveratrol; **1-1**, **1-2**, **1-3**, **1-4**: proposed products of resveratrol against ^1^O_2_). (**B**): Proposed mechanism of pterostilbene quenching ^1^O_2_ (**2**: pterostilbene; **2-1**, **2-2**: proposed products of pterostilbene against ^1^O_2_). (**C**): Proposed mechanism of piceatannol quenching ^1^O_2_ (**3**: piceatannol; **3-1**, **3-2**, **3-3**: proposed products of piceatannol against ^1^O_2_.).

*Trans*-resveratrol (**1**), *trans*-pterostilbene (**2**) and *trans*-piceatannol (**3**) all have stilbene skeletons, while their substituents are different. As shown in Figure 1, *Trans*-resveratrol contains a resorcinol ring and a phenol ring, *trans*-pterostilbene contains a 3,5-dimethoxybenzene ring and a phenol ring, *trans*-piceatannol contains a catechol ring and a phenol ring. In order to investigate the characteristic structure and the mechanism of stilbenes quenching ^1^O_2_, we performed qualitative and quantitative analysis of reactants and products in the three stilbenes quenching ^1^O_2_ by using UHPLC-QTOF-MS^2^ and UHPLC-QQQ-MS^2^, and the B3LYP density functional method was used to verify the proposed mechanism.

## 2. Materials and Methods

### 2.1. Reagents and Sample Preparation

Reagents: Methanol used for UHPLC analysis was purchased from Thermo Fisher Scientific (Waltham, MA, USA). Deionized water was purified with a Milli-Q water system (Millipore, Bedford, MA, USA). *Trans*-resveratrol (purity > 98%), *trans*-pterostilbene (purity > 97%) and *trans*-piceatannol (purity > 98%) were acquired from Sigma-Aldrich (St. Louis, MO, USA). Rose Bengal (RB) was purchased from Beijing Yaanda Biotechnology Co., Ltd. (Beijing, China). As a photosensitizer, RB can absorb energy from light then transfer the energy to molecular oxygen and generating ^1^O_2_.

Sample preparation: All experiments involving the application of *trans*-resveratrol, *trans*-pterostilbene and *trans*-piceatannol were carried out in a dimly lit environment to prevent photoisomerisation [23]. The concentration of RB dissolved in deionized water during sample preparation was 18 μmol/L. Both the use and storage of RB need to be performed in a dimly lit environment. The concentration of stilbenes dissolved in methanol was 500 μmol/L. Prepared solutions need to be filtered by 0.22 μm filter. The ultraviolet lamp (UVA, 20 W, λ = 365 W) was turned on for at least 15 min to reach an equilibrium status. Each control sample contained 500 μL ultrapure water and 100 μL *trans*-stilbenes (**1**, **2**, **3**, respectively) without ultraviolet radiation (**S1**, **S2** and **S3**). Treated samples were composed of 500 μL RB and 100 μL *trans*-stilbenes (**1**, **2**, **3**, respectively); the reaction mixtures were irradiated by the ultraviolet lamp for 2 min at 23 °C ± 2 °C (**S4**, **S5** and **S6**). All samples were freshly prepared and immediately determined and analyzed after transferring to the auto-sampler vials.

### 2.2. Density Functional Method

The B3LYP density functional method was employed in this study to carry out the computations. The 6-311g(d,p) basis set was used for all the atoms in geometry optimizations. Vibrational frequency analyses at the same theoretical level were performed on all optimized structures to characterize stationary points as local minima. Only the energy of conformation most stable for compounds was used to compare the stability of possible products. The Gaussian 09 suite of programs was used to compute the energy of possible product (with the aid of literature, compounds **1-1**, **1-2** were selected as the possible product of resveratrol quenching ^1^O_2_).

### 2.3. Qualitative Analysis by UHPLC-QTOF-MS^2^

The preparation of the sample was described in Section 2.2. The UHPLC-QTOF-MS^2^ experiments were carried out by a Thermo Scientific Dionex UltiMate 3000 system coupled with a Bruker micrOTOF-Q III mass spectrometer (Bruker-Franzen Analytik GmbH, Bremen, Germany). The separation of samples was performed on a Thermo Scientific Acclaim^TM^ RSLC 120 C_18_ reversed-phase column (3.0 × 100 mm, 2.2 μm, 120 Å), using a gradient elution comprising Ammonium formate in Ultra-pure water (5 mmol/L) and methanol with a flow rate of 0.2 mL/min at 20 °C. The injection volume was 2 μL. The separation was achieved using multi-step gradient using solvent A (water) and solvent B (methanol). The percentage of B (methanol) increased linearly from 5% to 30% in the first 5 min. Afterwards, it increased to 50% during the next 5 min. Then, it increased to 90% in 5 min and held for 5 min. The percentage of B went back to 5% in 7 min, and held for 5 min. The total run-time was 32 min.

The induction of UHPLC effluent introduced into the ESI source by a solvent line (analytical, softron P/N 5040.8117). Software HyStar3.2 (Bruker Hyphenation Star Application, Hamburg, Germany) was used to combine control of UHPLC and MS. Experiments were performed under the negative ion mode of ESI. Nitrogen was used as the nebulizing and drying gas at 1.2 bar and a flow rate of 8.0 L/min, with the dry temperature set as 180 °C. Capillary voltage was 3000 V, and End Plate Offset was 500 V. The scan mode was set to Auto MS/MS with the mass scan range being 50–1000 *m*/*z*.

### 2.4. Quantitative Analysis by UHPLC-QQQ-MS^2^

The preparation of the sample has been described in Section 2.2. The UHPLC-QQQ-MS^2^ system from Agilent Technologies (Santa Clara, California) consisted of a 1260-series UHPLC coupled to an Agilent 6460 series triple quadrupole mass spectrometer. Sample separation was performed on an Agilent Poroshell 120 EC-C_18_ column (3.0 × 100 mm, 2.7 μm), using a gradient elution including the ultra-pure water with 5 mmol/L ammonium formate added (solvent A) and MeOH (solvent B) with a flow rate of 0.2 mL/min at 30 °C and the injection volume was 2 μL. The separation was achieved using multi-step gradient program as follows: 0–2 min, linearly 50% B–60% B; 2–3 min, linearly 60% B–70% B; 3–5 min, linearly 70% B–80% B; 5–12 min, 80% B; 12–14 min, linearly 80% B–90% B; 14–16 min, linearly 90% B; 16–20 min, linearly 90% B–50% B; 20–23 min, 50% B. The total running time was 23 min.

Mass spectrometric detection was performed on an Agilent 6460 QQQ instrument equipped with the Agilent Jet Stream Technologies - Electrospray Ionization (AJS-ESI) source operating in a negative ionization mode, with the conditions as follows: Nitrogen (99.99%) was used as the nebulizing and drying gas at 45 psi and a flow rate of 5.0 L/min, with the drying temperature set as 350 °C; the temperature of sheath gas was 250 °C, and the flow was 11 L/min; the capillary voltage was 3500 V; and the nozzle Voltage was 500 V. Agilent Mass Hunter software was used to control the UHPLC-QQQ-MS^2^. Multiple Reaction Monitoring (MRM) mode with optimal Fragmentor (V) and Collision Energy (eV) were used to quantify three stilbenes. MRM mode was also used to quantify reactants. The optimal parameters used in MRM mode are shown in Table 1. It is generally accepted that peak area represents the relative content of target compounds. The ratio of the change of reactant content to the initial value of reactant, namely, the relative content change of reactants, was used to evaluate the activity of stilbene quenching ^1^O_2_.

### 2.5. Statistical Analysis

Data were analyzed by Microsoft Excel 2016 and Statistical Analysis Software (SPSS18). Results were statistically compared and expressed as means with standard deviations (SD). The data were analyzed by one-way analysis of variance (ANOVA). Comparison of means was made by Duncan’s multiple range tests. Average values and standard errors are shown in the figures. Differences were considered significant when *p* < 0.05.

## 3. Results and Discussion

### 3.1. Qualitative Comparison of the Activity of Double Bond and Resorcinol Ring Quenching ^1^O_2_

**Figure 2:**
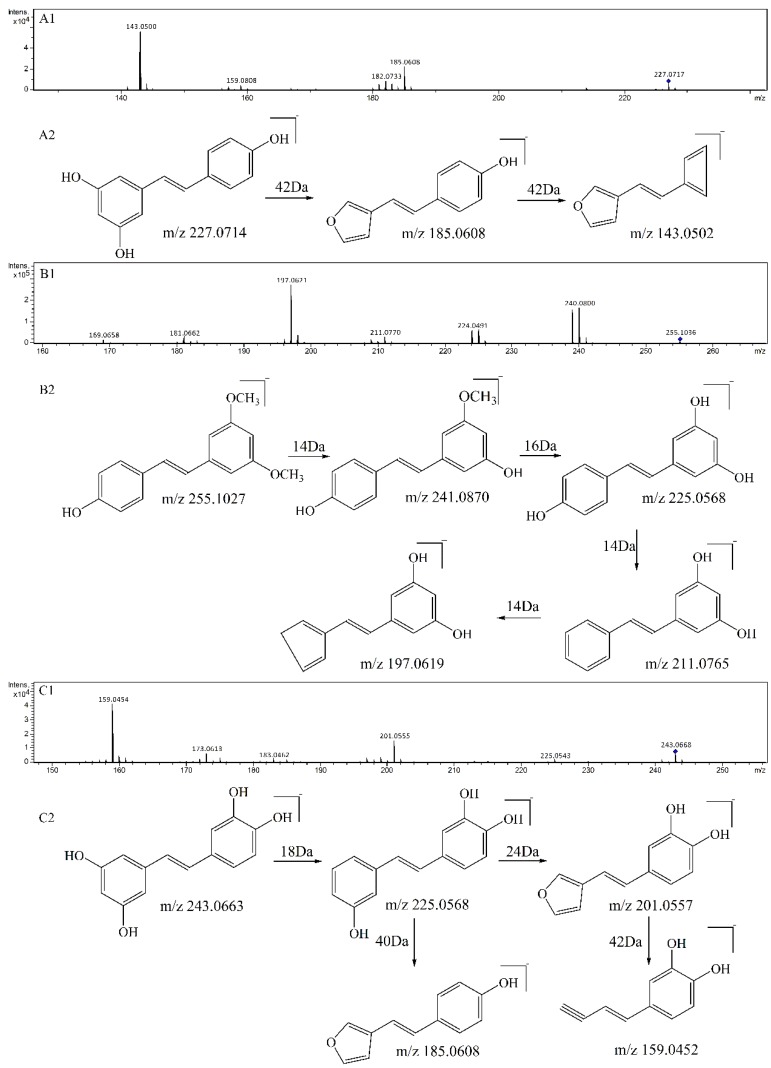
MS^2^ spectrum of the precursor ion at *m*/*z* 227 (**A1**) and its proposed fragmentation pathway (**A2**). MS^2^ spectrum of the precursor ion at *m*/*z* 255 (**B1**) and its proposed fragmentation pathway (**B2**). MS^2^ spectrum of the precursor ion at *m*/*z* 243 (**C1**) and its proposed fragmentation pathway (**C2**).

**Figure 3:**
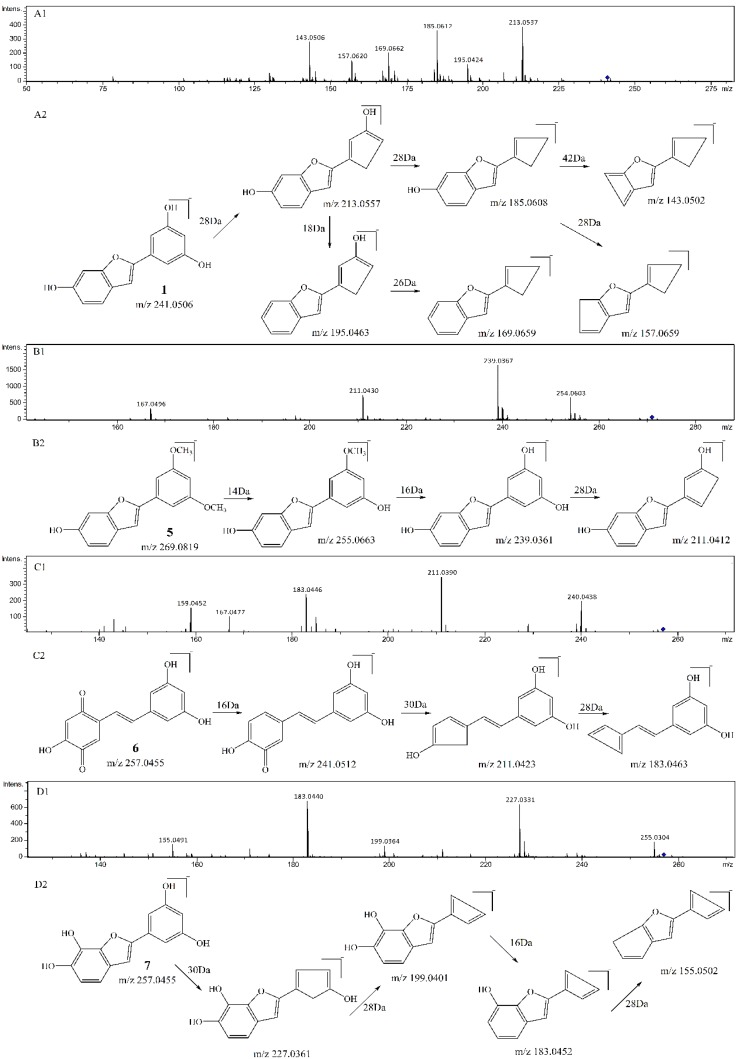
MS^2^ spectrum of the precursor ion at *m*/*z* 241 (**A1**) and its proposed fragmentation pathway (**A2**). MS^2^ spectrum of the precursor ion at *m*/*z* 269 (**B1**) and its proposed fragmentation pathway (**B2**). MS^2^ spectrum of the precursor ion at *m*/*z* 257^a^ (**C1**) and its proposed fragmentation pathway (**C2**). MS^2^ spectrum of the precursor ion at *m*/*z* 257^b^ (**D1**) and its proposed fragmentation pathway (**D2**).

As compared with sample **S1** (resveratrol), the relative abundance of *m*/*z* 227.0717 in sample S4 (resveratrol + ^1^O_2_) decreased sharply. The product ions of *m*/*z* 227.0717 were *m*/*z* 185, 159, 143, and its proposed fragmentation pathway is shown in Figure 2A. In the MS/MS spectrum of the [M − H]^−^ ion at *m*/*z* 241.0528, it generated losses of 28 Da, 46 Da, 56 Da, 72 Da, 84 Da, 98 Da, corresponding to the product ions at *m*/*z* 213, 195, 185, 169, 157, and 143. Its proposed fragmentation pathway is shown in Figure 3A. The precursor ions at *m*/*z* 121.0287, *m*/*z* 137.0244 and *m*/*z* 241.0528 were detected in sample **S4**, but not in sample **S1**. It is noteworthy that 241.0528 was 14 Da larger than that of resveratrol (**1**, *m*/*z* 227.0717). The ions at *m*/*z* 241.0528 and *m*/*z* 227.0717 had similar fragmentation pathways. In other words, they have the same product ions such as *m*/*z* 185, 159, 143. Celaje et al. (2011) reported that resveratrol could quench ^1^O_2_ and generate p-hydroxybenzaldehyde, 3,5-dihydroxybenzaldehyde and Moracin M, proving that ions at *m*/*z* 121.0295 (**1-3**), *m*/*z* 137.0244 (**1-4**), and *m*/*z* 241.0506 (**1-1**) were the oxide products of resveratrol quenching ^1^O_2_ [21]. According to their work, the characteristic structure in the process of resveratrol quenching ^1^O_2_ is the carbon-carbon double bond. However, Jiang et al. (2010) reported that resveratrol quinone (**1-2**) was the main product responsible for quenching ^1^O_2_ and suggested that what really matters in the mechanism is the resorcinol moiety [20]. In addition, according to our previous result [22], the carbon-carbon double bond and resorcinol moiety both were verified to be participants in the quenching reaction.

As compared with sample **S2** (pterostilbene), the relative abundance of *m*/*z* 255.1036 in sample V (pterostilbene + ^1^O_2_) decreased significantly. The product ions of *m*/*z* 255.1036 were *m*/*z* 240, 225, 211, 197, and its proposed fragmentation pathway is shown in Figure 2B. In the MS/MS spectrum of the [M − H]^−^ ion at *m*/*z* 269.0815, it generated the losses of 14 Da, 30 Da, 58 Da, corresponding to the product ions at *m*/*z* 255, 239, 211. The precursor ions at *m*/*z* 121.0290 and *m*/*z* 269.0815 were detected in sample **S5**, but not in sample **S2**. It is worthwhile mentioning that *m*/*z* 269.0815 was14 Da larger than that of pterostilbene (**2**, *m*/*z* 255.1036). The content of pterostilbene decreased (Figure 3B), and ions at *m*/*z* 269.0815 and *m*/*z* 255.1036 had similar fragmentation pathways. Compounds **2-1**, **2-2** could be the oxide products of pterostilbene quenching ^1^O_2_.

As compared with sample **S3** (piceatannol), the relative abundance of *m*/*z* 243.0668 in sample VI (piceatannol + ^1^O_2_) decreased sharply. The product ions of *m*/*z* 243.0668 were *m*/*z* 225, 201, 185, 159, and its proposed fragmentation pathway is shown in Figure 2C. The precursor ions at *m*/*z* 137.0256 and *m*/*z* 257 were detected in sample **S6**, but not in sample **S3**. The precursor ions at *m*/*z* 257 were observed at different retention times, corresponding to **3-1** (*m*/*z* 257.0477^a^, T = 13.7 min) and **3-2** (*m*/*z* 257.0460^b^, T = 15.1 min). In the MS/MS spectrum of the [M − H] ^−^ ion at *m*/*z* 257.0477^a^ (**3-1**), the mass spectrum generated losses of 16 Da, 46 Da and 74 Da, which correspond to the product ions at *m*/*z* 241, 211, 183. The proposed fragmentation pathway of *m*/*z* 257.0477^a^ is shown in Figure 3C. In the MS/MS spectrum of the [M − H]^−^ ion at *m*/*z* 257.0460^b^ (**3-2**, T = 15.1 min), the mass spectrum generated losses of 30 Da, 58 Da, 74 Da and 102 Da, which correspond to the product ions at *m*/*z* 227, 199, 183, 155. The proposed fragmentation pathway of *m*/*z* 257.0460^b^ is shown in Figure 3D. The precursor ions at 257 were only observed in sample **S6**, and their fragmentation pathways are similar to *m*/*z* 243.0668. The precursor ion at 257 is considered a product of piceatannol quenching ^1^O_2_.

When ^1^O_2_ was added to the reaction, the content of resveratrol, pterostilbene and piceatannol decreased significantly. The identical substructure of the three compounds is the stilbene skeleton, pointing out the carbon-carbon double bond may be the characteristic structure of stilbenes quenching ^1^O_2_. We suggest the main mechanism of stilbenes quenching ^1^O_2_ could be the [2+2] and [4+2] cycloaddition reaction of the carbon-carbon double bond in stilbenes.

### 3.2. Quantitative Comparison of the Activity of the Catechol Ring, Carbon-Carbon Double Bond and Resorcinol Ring Quenching ^1^O_2_

**Figure 4:**
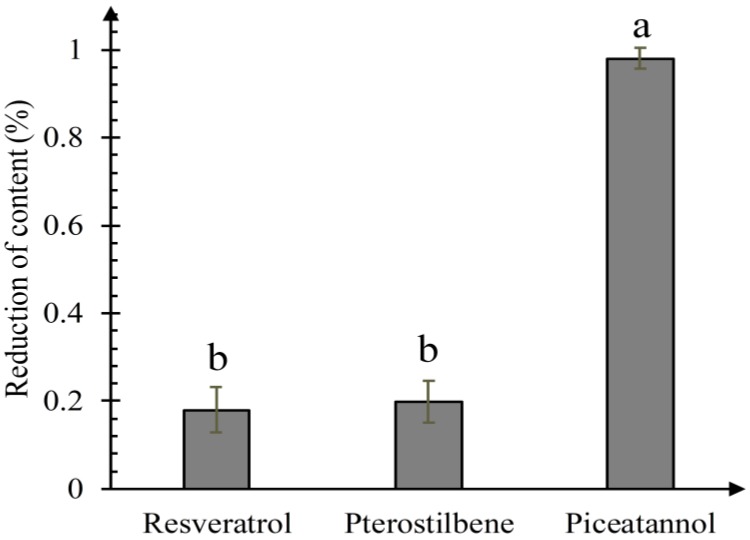
Changes of three stilbene contents in the prossess of quenching ^1^O_2_. The values are means of three replicates and their standard errors. Means with different letters are significantly different according to Duncan’s multiple-range test (*p* < 0.05).

As shown in Figure 4, the relative content of three stilbenes decreased differently. Piceatannol decreased most significantly, and there was no significant difference between resveratrol and pterostilbene reduction. Quantitative results showed that piceatannol had the strongest activity of quenching ^1^O_2_, followed by resveratrol and pterostilbene. Methoxylation of hydroxyl groups did not lead to any significant decrease of the^1^O_2_ quenching activity, further proving that cycloaddition reaction of the carbon-carbon double bond plays an important role in quenching ^1^O_2_, and it is more effective than the resorcinol ring. Several articles reported that the carotenoids have a strong activity of quenching ^1^O_2_, closely related to the existence of conjugated double bonds [24,25]. Pallidol has the strong ability to quench ^1^O_2_ [15], but it does not contain any carbon-carbon double bond, only resorcinol rings. We propose that the carbon-carbon double bond is the characteristic functional group in stilbenes quenching ^1^O_2_. However, if there is no carbon-carbon double bond in the compound, the resorcinol ring may participate in the reaction of quenching ^1^O_2_, generating quinones. Furthermore, the catechol ring has been proposed to be the most vital functional group of quenching ^1^O_2_. Our studies showed that piceatannol was the most involved in the reaction. Jung et al. (2010) determined that the total singlet oxygen quenching rate constant for resveratrol in methanol was 2.55 × 10^7^ M^−1^ s^−1^ [8]. Choi et al. (2016) determined that the total singlet oxygen quenching rate constant for nordihydroguaiaretic acid (NDGA) in methanol was 9.81 × 10^7^ M^−1^ s^−1^ [26]. NDGA contains two catechol groups, while resveratrol contains double bond and resorcinol rings. NDGA has a stronger singlet oxygen quenching activity than resveratrol, indicating that catechol ring was more effective than the carbon-carbon double bond in resorcinol’s quenching of ^1^O_2_. Therefore, we conclude that the most important substructure of quenching ^1^O_2_ is the catechol ring, then the carbon-carbon double bond, and then the resorcinol ring.

### 3.3. Validation of the Mechanism by B3LYP Density Functional Method

According to literature, compounds **1-1** and **1-2** were considered as the possible products of **1** quenching ^1^O_2_. In order to determine which product is more effective in resveratrol quenching ^1^O_2_, we used the B3LYP density functional method to determine the optimal configurations and computed the energy of two compounds. The parameters of the optimal configurations and energy of compounds **1-1** and **1-2** are shown in Table 2. The energy calculated by the B3LYP density functional method is E_**1-1**_(b3lyp/6-311g(d,p)) = −840.6133 Hartree/Particle < E_**1-2**_(b3lyp/6-311g(d,p)) = −840.5894 Hartree/Particle. It is widely accepted that compounds with lower energy have higher stability [27], which in our case shows that compound **1-1** is more stable and more likely to generate in theory. Compound **1-1** is the possible product of resveratrol quenching ^1^O_2_, pointing to the carbon-carbon double bond as the important structure responsible in resorcinol’s quenching of ^1^O_2_. The bond length and bond angle of compound 1-1, 1-2 are shown in Table S1.

## 4. Conclusions

The main mechanism of resveratrol quenching ^1^O_2_ was the [2+2] and [4+2] cycloaddition reaction of the carbon-carbon double bond. Furthermore, the catechol ring, carbon-carbon double bond and resorcinol ring in stilbenes also participated in quenching ^1^O_2_, and the activity of structure responsible for quenching ^1^O_2_ decreased in the order: catechol ring > carbon-carbon double bond > resorcinol ring. The mechanism could provide a theoretical basis for future screening and drug design for further investigations of those compounds’ applications in ^1^O_2_-mediated diseases.

## Figures and Tables

**Table 1:** The optimal parameters of target compounds in MRM mode.

Compounds	Precursorion	Production	Fragmentor (V)	Collision Energy (eV)
resveratrol	*m*/*z* 227	*m*/*z* 185	75	15
pterostilbene	*m*/*z* 255	*m*/*z* 239.8	75	15
piceatannol	*m*/*z* 243	*m*/*z* 200.6	90	8

**Table 2:** The E(b3lyp/6-311g(d,p)) of compound 1-1, 1-2 and parameters of the optimal configurations.

Compound 1-1	Compound 1-2
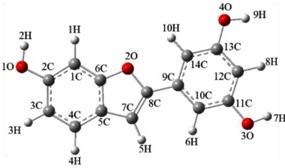	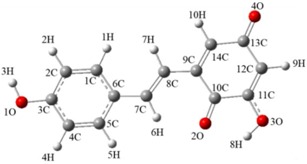
E(b3lyp/6-311g(d,p))	
−840.6133 Hartree/Particle	−840.5894 Hartree/Particle

## Supplementary Materials

The following are available online at https://www.mdpi.com/2218-273X/9/7/268/s1, Table S1. The bond length and bong angle of compound **1-1**, **1-2** and parameters of the optimal configurations.
