# Supplementary material for: Carbon-Carbon Double Bond and Resorcinol in Resveratrol and Its Analogues: What Is the Characteristic Structure in Quenching Singlet Oxygen?
Source: Biomolecules. 2019 Jul 9;9(7):268. doi: 10.3390/biom9070268 (PMC6681369; doi:10.3390/biom9070268)
Supplement: Supplementary file 1 [file biomolecules-09-00268-s001.pdf]

Table S1. The bond length and bong angle of compound 1-1, 1-2 and parameters of the optimal configurations.

**compound 1-1**				**compound 1-2**			
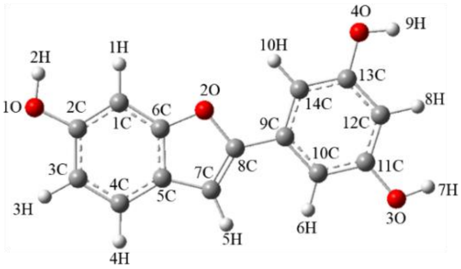				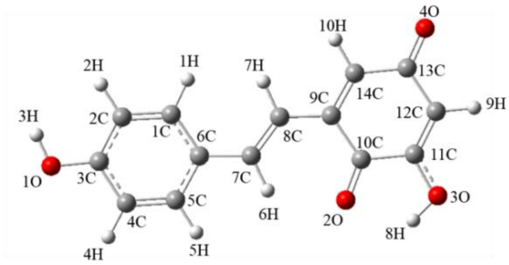			
E(b3lyp/6-311g(d,p))							
-840.6133 Hartree/Particle				-840.5894 Hartree/Particle			
Bond length (A°)		Bond angle (°)		Bond length (A°)		Bond angle (°)	
1C-2C	1.394	2C-1C-1H	122.225	1C-1H	1.083	6C-1C-1H	120.21
1C-1H	1.084	2H-1O-2C	109.362	1C-2C	1.386	6C-1C-2C	121.339
2C-3C	1.409	1O-2C-1C	122.323	2C-2H	1.086	1C-2C-2H	119.98
2C-1O	1.367	1C-2C-3C	121.227	2C-3C	1..4	1C-2C-3C	120.189
1O-2H	0.963	2C-3C-3H	117.804	3C-1O	1.361	2C-3C-1O	122.738
3C-4C	1.384	2C-3C-4C	121.041	1O-3H	0.963	2C-3C-4C	119.61
3C-3H	1.083	3C-4C-4H	120.055	3C-4C	1.4	3C-1O-3H	109.568
4C-5C	1.403	4H-4C-5C	120.881	4C-4H	1.083	3O-4C-4H	118.931
5C-6C	1.404	4C-5C-7C	136.337	4C-5C	1.385	3C-4C-5C	119.622
6C-1C	1.388	4C-5C-6C	118.328	5C-5H	1.084	4C-5C-5H	119.12
6C-2O	1.361	5C-6C-1C	123.948	5C-6C	1.408	4C-5C-6C	121.972
5C-7C	1.436	5C-6C-2O	110.343	6C-1C	1.406	5C-6C-7C	118.62
7C-8C	1.364	5C-7C-8C	107.032	6C-7C	1.458	5C-6C-1C	117.269
7C-5H	1.078	5C-7C-5H	126.988	7C-6H	1.083	6C-7C-6H	115.295
8C-2O	1.387	7C-8C-9C	133.091	7C-8C	1.351	6C-7C-8C	126.555
2O-6C	1.361	7C-8C-2O	110.432	8C-7H	1.087	7C-8C-7H	119.018
8C-9C	1.457	8C-2O-6C	106.859	8C-9C	1.453	7C-8C-9C	128.017

9C-10C	1.403	8C-9C-14C	120.093	9C-10C	1.492	8C-9C-14C	120.656
10C-11C	1.389	8C-9C-10C	119.883	10C-2O	1.223	8C-9C-10C	122.685
10C-6H	1.082	9C-10C-6H	121.835	10C-11C	1.506	9C-10C-2O	124.905
11C-12C	1.398	9C-10C-11C	119.65	11C-3O	1.337	9C-10C-11C	118.851
11C-3O	1.366	10C-11C-12C	120.767	3O-8H	0.976	10C-11C-3O	113.794
3O-7H	0.962	10C-11C-3O	117.211	11C-12C	1.345	10C-11C-12C	122.152
12C-13C	1.398	11C-3O-7H	109.461	12C-9H	1.082	11C-3O-8H	105.828
12C-8H	1.087	11C-12C-13C	119.172	12C-13C	1.466	11C-12C-9H	122.141
13C-14C	1.396	11C-12C-8H	120.359	13C-4O	1.225	11C-12C-13C	119.678
13C-4O	1.366	12C-13C-4O	122.089	13C-14C	1.48	12C-13C-4O	122.031
4O-9H	0.962	12C-13C-14C	120.8	14C-10H	1.085	12C-13C-14C	118.099
14C-9C	1.4	13C-4O-9H	109.472	9C-14C	1.359	13C-14C-10H	114.474
14C-10H	1.08	13C-14C-10H	119.341			13C-14C-9C	120.875
6C-1C-1H	121.383	13C-14C-9C	119.587				
